# The effect of sodium butyrate and cisplatin on expression of EMT markers

**DOI:** 10.1371/journal.pone.0210889

**Published:** 2019-01-17

**Authors:** Alena Mrkvicova, Marcela Chmelarova, Eva Peterova, Radim Havelek, Ivana Baranova, Petra Kazimirova, Emil Rudolf, Martina Rezacova

**Affiliations:** 1 Department of Medical Biochemistry, Faculty of Medicine in Hradec Kralove, Charles University, Hradec Kralove, Czech Republic; 2 Institute for Clinical Biochemistry and Diagnostics, Faculty of Medicine in Hradec Kralove, Charles University and University Hospital Hradec Kralove, Hradec Kralove, Czech Republic; 3 Second Department of Internal Medicine – Gastroenterology, Faculty of Medicine in Hradec Kralove, Charles University and University Hospital Hradec Kralove, Hradec Kralove, Czech Republic; 4 Department of Biology, Faculty of Medicine in Hradec Kralove, Charles University, Hradec Kralove, Czech Republic; University of South Alabama Mitchell Cancer Institute, UNITED STATES

## Abstract

Histone modifications play a key role in the epigenetic regulation of gene transcription in cancer cells. Histone acetylations are regulated by two classes of enzymes, histone acetyltransferases (HATs) and histone deacetylases (HDACs). HDACs are increased in ovarian carcinomas and they are involved in carcinogenesis and resistance to chemotherapeutic agents. In our study we investigated anticancer effect of HDAC inhibitor sodium butyrate (NaBu) on cisplatin-sensitive and cisplatin-resistant ovarian cell lines A2780 and A2780cis. A2780 and A2780cis were treated with NaBu alone or in combination with cisplatin (CP). NaBu inhibited the growth of both cell lines and enhanced cytotoxic effect of CP. Exposure to NaBu for 24 h induced cell cycle arrest. The expressions of EMT-related genes and proteins were further investigated by qPCR and western blot analysis. Loss of E-cadherin has been shown to be crucial in ovarian cancer development. We found that NaBu dramatically induce expression of E-cadherin gene (*CDH1*) and protein levels in A2780 and A2780cis. We investigated correlation between transcription and methylation of *CDH1*gene. Methylation level analysis in 32 CpG sites in *CDH1* gene (promoter/exon1 regions) was performed using bisulfite NGS (Next Generation Sequencing). We found that cisplatin-resistant cell line A2780cis cells differ from their cisplatin-sensitive counterparts in the *CDH1* methylation. Methylation in A2780cis cells is elevated compared to A2780. However, NaBu-induced expression of CDH1 was not accompanied by CDH1 demethylation. NaBu treatment induced changes in expression of EMT-related genes and proteins. Interestingly E-cadherin zinc finger transcriptional repressor *SNAIL1* was upregulated in both cell lines. Mesenchymal marker vimentin was downregulated. Matrix metalloproteases (MMPs) are necessary for pericellular proteolysis and facilitate migration and invasion of tumour cells. NaBu induced mRNA expression of MMPs, mild changes in activities of gelatinases MMP2 and MMP9 were detected. Our data demonstrate that NaBu sensitizes cisplatin-resistant ovarian cancer cells, re-established E-cadherin expression, but it was not able to reverse the EMT phenotype completely.

## Introduction

Ovarian cancer is the leading cause of death from gynecologic tumors. Bad prognosis of the disease is attributed to its aggressive nature and the fact that the majority of cases are diagnosed in advanced stages accompanied by intraperitoneal metastatic dissemination [1]. Besides genetic alterations epigenetic regulation (DNA methylation and histone modifications) play significant role in the cancer progression. DNA methylation is mediated by DNA methyltransferases, which catalyze the covalent addition of a methyl group to the 5-carbon of the cytosine in CpG context dispersed throughout the genome or in DNA repetitive regions. Promoter DNA methylation at CpG sites represses gene expression by impeding access to transcription factors and inhibiting RNA polymerase II [2]. Histone modifications are regulated by two classes of enzymes, histone acetyltransferases (HATs) and histone deacetylases (HDACs). HDACs are often overexpressed in cancer cells, resulting in histone hypoacetylation and repression of numerous genes. Overexpression of HDAC 1, 2 and 3 has previously been reported in ovarian cancer tissues [3].

Platinum compounds alone or in combination with paclitaxel constitute the most active and standard chemotherapy treatment of ovarian cancer. Unfortunately, majority of patients relapse after treatment. About 20% of all ovarian cancer relapses are platinum-refractory with very poor prognosis [4]. Acquired drug resistance has been studied in several types of cisplatin-resistant cell lines. Multiple molecular mechanisms including impaired intracellular drug accumulation or DNA damage response were identified [5]. Recent studies also suggest a role for DNA methylation and histone modifications in drug resistance as reviewed in [6]. These findings make epigenetic changes an attractive therapeutic target.

Recent evidence suggests that HDAC inhibitors (HDACi) re-induce histone acetylation and thus regulate cell growth, apoptosis and cell differentiation in many types of cancer. For instance HDACi induced apoptosis and autophagy in pancreatic cancer cell [7], decreased ovarian cancer cell motility and caused re-expression of tumor suppressor genes [8]. It was also demonstrated that HDACi sensitizes cancers cells to cisplatin (CP) [9].

One of the oncogenic mechanisms that are under epigenetic control and may be affected by HDACi is epithelial—mesenchymal transition (EMT). EMT is a complex process by which polarized epithelial cells acquire mesenchymal phenotype through a loss of epithelial cell—cell junction and actin cytoskeleton reorganization [10]. Cells undergoing EMT display decreased expression of epithelial markers such as E-cadherin (encoded by *CDH1* gene) and zona occludens protein 1 (ZO-1). Furthermore, morphological changes are accompanied with N-cadherin (encoded by *CDH2* gene) and vimentin expression which is typical for completed mesenchymal interchange. EMT is driven by SNAIL, SLUG, and zinc-finger E-box binding (ZEB) transcription factors, which downregulate tight junction proteins claudin and occludin [11] [12] as well as E-cadherin. During last years it became apparent that epigenetic regulation of gene expression is crucial during EMT, although the exact mechanisms of interplay between histone acetylations and altered expression of EMT proteins are far from understood. Zinc finger transcriptional repressor SNAIL1 was found to be associated with HDAC1/2 and other proteins in multimolecular complex to mediate repression of *CDH1* promoter [13]. However, the relationship between epigenetic histone modifications and EMT of ovarian cancer cells remains controversial.

In this study we investigated the effect of HDACi sodium butyrate (NaBu) alone or in combination with CP on EMT of ovarian cancer cells line A2780 and cisplatin-resistant subclone A2780cis. We found that NaBu inhibits cell cycle progression and potentiates CP cytotoxicity. We observed restoration of E-cadherin expression in both cells lines, but also massive upregulation of SNAIL and matrix metalloproteinases (MMPs) after NaBu treatment. Finally, we identified higher methylation in *CDH1* gene in chemoresistant ovarian cancer cells compared to the chemosensitive ovarian cancer cells. Despite restoration of E-cadherin expression, NaBu treatment did not show significant effect on *CDH1* methylation.

## Materials and methods

### Cell culture and treatment

Human ovarian cancer cell lines A2780 and A2780cis were obtained from European Collection of Animal Cell Cultures (Porton Down, England) and grown in RPMI 1640 (Sigma-Aldrich, St. Louis, USA) containing 10% fetal bovine serum (FBS) (Biowest, Miami, USA), 100 U/mL penicillin (Sigma-Aldrich, St. Louis, USA). Cells were maintained at 37°C in 5% CO_2_. In order to retain resistance to CP—cis-Diammineplatinum(II) dichloride (Sigma-Aldrich, St. Louis, USA) (1 μM) was added to the media every 2–3 passages. At all events the stock solutions were prepared fresh by dissolving 3 mg of CP in 10 mL cultivation medium to reach 1 mM concentration. Sodium butyrate (Sigma-Aldrich, St. Louis, USA) was dissolved into sterile PBS solution and diluted to working concentration 5 mM before use.

### Growth inhibition assay

The WST-1 (Roche, Mannheim, Germany) reagent was used to determine the effect of NaBu and CP. Cells were plated at 10 x 10^3^ cells per well in 96-well plates and incubated at 37 °C for 24 h. Both cell lines were treated with CP at concentrations ranging from 0–160 μM alone or in combination with NaBu. After a 48-h incubation period 50 μL WST-1 reagent was added. The absorbance was measured after 2 hours incubation at 440 nm and reference wavelength 690 nm. The measurement was performed in microplate reader (Epoch, BioTek, USA). All experiments were performed in three biological replicates. The IC_50_ values for each cell line was calculated.

### Measurement of cell proliferation

The cells were seeded at a concentration 15, 10 or 5 x 10^3^ cells/well in 150 μL culture medium. After 24 h cells were treated with NaBu (5 mM) or CP (5 μM) or combination of both. WST-1 reagent (50 μL) was added after another 24, 48 and 72 h. The absorbance was measured after 2 h incubation as described above.

### Cell cycle distribution

After 24 h of NaBu treatment the cells were washed with ice cold PBS and fixed with 70% (v/v) ethanol. In order to detect low molecular-weight fragments of DNA, the cells were incubated for 5 minutes at room temperature in a buffer (192 mL 0.2 M Na_2_HPO_4_ + 8 mL of 0.1 M citric acid, pH 7.8) and then labeled with propidium iodide in Vindelov’s solution for 1 h at 37 °C. The DNA content was determined using a CyAn flow cytometer (Beckman Coulter, Miami, FL, USA) with an excitation wavelength of 488 nm. The data were analyzed using Multicycle AV software (Phoenix Flow Systems, San Diego, CA, USA).

### RNA isolation, RT and qRT-PCR

The cells were washed with PBS, harvested and dissolved in lysis buffer. Total cellular RNA was extracted by RNeasy Mini kit according to manufacturer instructions (Qiagen, Hilden, Germany). 1 μg of RNA was reverse transcribed using cDNA Reverse Transcription Kit and quantified with TaqMan Gene Expression Assays. The assay ID numbers used in this study were CDH1 (Hs01023894_m1), CDH2 (Hs00983056_m1), CDKN1A (Hs00355782_m1), CTNNB (Hs00355049_m1), MMP1 (Hs00899658_m1), MMP2 (Hs01548727_m1), MMP9 Hs00957562_m1, SNAIL1 (Hs00195591_m1), SNAIL2 (Hs00950344_m1), VIM (Hs00185584_m1), ZEB1 (Hs00232783_m1), ZEB2 (Hs00207691_m1), ITGA2 (Hs00158127_m1), TIMP1 (Hs99999139_m1), TIMP2 (Hs00234278_m1) and HPRT (Hs02800695_m1), which was used as a reference gene. The relative expression was measured using Quantstudio 6 Real-Time PCR system (all obtained from Applied Biosystems, CA). mRNA levels were calculated using the comparative Ct Method (ΔΔCt method).

### Western blot analysis

The cells were harvested, suspended in PBS containing 4 mM EDTA and washed 3 times. The proteins were extracted with Cell lysis buffer (Cell Signaling Technology, Danvers, USA). Protein content was determined by Bicinchoninic Acid Protein Assay Kit (Sigma-Aldrich, St. Louis, USA). Forty-five μg of protein were applied on Novex NuPAGE 4–12% Bis-Tris gel (Invitrogen Life Technologies, Prague, Czechia) under nonreducing conditions. The proteins were transferred to 0.2 μm Hybond nitrocellulose membrane (GE Healthcare, München, Germany). The membranes were incubated with the corresponding primary antibodies overnight at 4 °C followed by the relevant secondary antibody for 1 h at room temperature. The antibody against total or acetylated histone H3 and EMT related primary and secondary antibodies were (included in Epithelial-Mesenchymal Transition Antibody Sampler Kit) were obtained from Cell Signalling Technology (Danvers, MA, USA). Anti p21 antibody was purchased from (Sigma-Aldrich, St. Louis, USA). Staining with primary monoclonal mouse anti-βactin (Sigma-Aldrich, St. Louis, USA) was used as a loading control. The goat anti-mouse secondary antibody and detection system Western Blotting Luminol Reagent were purchased from Santa Cruz Biotechnology (Dallas, USA). The blots were scanned using PXi imaging system (Syngene, Cambridge UK).

#### Gelatin zymography

Cells were treated with NaBu (5 mM) or CP (5 μM) or combination of both. Next day medium was replaced with serum free medium. The medium was collected after 24 h and protein concentration was measured. Samples (5 mg per well) were electrophoresed in 8% SDS-polyacrylamide gel containing 0.1% gelatin (Sigma-Aldrich, St. Louis, USA). The gel was washed in 2.5% Triton X-100 for 1 h and subsequently incubated for 28 h at 37°C in 50 mmol/L Tris-HCl buffer pH 7.4 containing 15 mmol/L sodium chloride and 10 mmol/L calcium chloride. The gel was stained with Coomasie brillant blue and destained with 40% methanol and 10% acetic acid solution. Gelatinolytic bands were size-calibrated with a high molecular mass marker (Bio-Rad Laboratories (Hercules, CA).

### Bisulfite Next Generation Sequencing of CDH1

DNA was extracted from cells using the Qiagen (Hilden, Germany) DNA extraction kit. 500 ng of genomic DNA was treated with bisulfite using the EZ DNA Methylation-Gold Kit according to the manufacturer’s protocol (Zymo Research Corporation, USA). The MiSeq sequencer was used to search for methylation level in selected CpG sites in CDH1 gene. All experiments were done in triplicates.

Specific primers were designed to amplify promoter/exon 1 regions of the CDH1 gene (MethPrimer design). Sequencing libraries were prepared using sequence-specific primers with adapters (see S1 Table) for the Multiplicom MID Illumina MiSeq kit (Multiplicom, Belgium). First PCR was carried out in a 20 μL mixture containing 10x Gold buffer without MgCl_2_ (2 μL), 25mM MgCl_2_ (2 μL), 2.5 mM dNTPs solution Takara (1.6 μL), 10 pmol/μl primers (1 μL), 5U/μL AmpliTaq Gold DNA Polymerase (0.3 μL) (Applied Biosystems, CA), water and 2 μL of bisulfite-converted DNA in the Veriti Thermocycler (Applied Biosystems, CA). The cycling condition consisted of an initial denaturation at 95 °C for 5 minutes, 35 cycles of denaturing at 95 °C for 20 seconds, annealing at 62 °C for 30 seconds, and extension at 72 °C for 35 seconds, followed by final extension for 5 minutes at 72 °C. Universal methylated and unmethylated DNA (Zymo Research Corporation, USA) was similarly treated with bisulfite and used as controls. PCR products from the first PCRs were purified using magnetic beads (SPRISelect, Beckman Coulter, USA), 300x diluted and amplified in a subsequent barcoding PCR (Multiplicom). PCR products were separated by electrophoresis on 2% agarose gels and specific products were purified using NucleoSpin Gel and PCR Clean-up (Macherey-Nagel, Germany). Purified sample concentrations were measured using the Qubit Fluorometr (Thermo Scientific, USA). All samples were equimolarly pooled into one library. The molarity of the library was quantified using the KAPA library quantification assay (Kapa Biosystems, USA) and 4 nM library was prepared. Next-generation sequencing was carried out on the Illumina MiSeq using Reagent Kit v2 with 250 base pair paired-end sequencing following the manufacturer’s instructions. Data were analyzed using NextGENe software (Softgenetics, USA). Bisulfite-converted methylated DNA was used as a reference sequence.

### Statistical analysis

Results are presented as mean ± SD. Student’s *t*-test was used to compare two groups consisting of normally distributed interval data. One-way ANOVA and Kruskal-Wallis tests were used to compare three or more groups when interval data was normally distributed and not necessarily normally distributed, respectively. All calculations were performed using GraphPad Prism version 6.00 for Windows, GraphPad Software (San Diego, California USA).

## Results

### Antiproliferative effect of sodium butyrate alone or in combination with cisplatin

**Fig 1:**
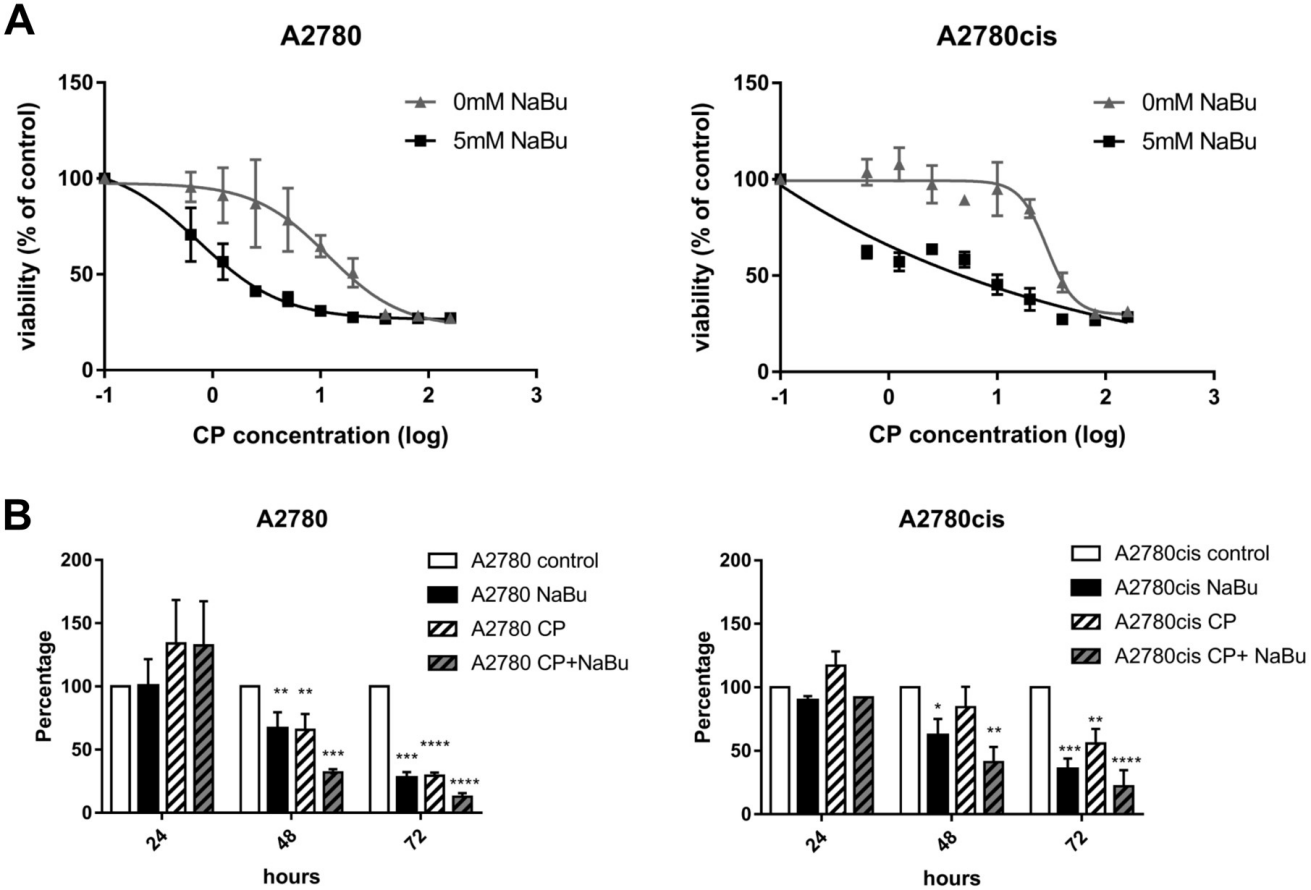
Sodium butyrate enhances the cytotoxic effect of cisplatin. Cell proliferation/viability was measured using colorimetric WST-1 assay. Data are presented as the percentage relative to untreated cells, arbitrary set to 100%. Dose response of A2780 and A2780cis cells to 48 h treatment with CP alone or in combination with NaBu (5 mM) (A). Each point represents the mean ± SD from 2 independent experiments. Dynamic of antiproliferative effect of NaBu (5 mM) alone and in combination with CP (5 μM) (B). The data points in the graph are the means ± SD of 3 independent experiments. Significance was tested using one-way ANOVA. *P ≤ 0.05, **P ≤ 0.01, *** P≤ 0.001, **** P≤ 0.0001 are significantly different from control values.

The effect of NaBu alone or in combination with CP on the viability and proliferation of A2780 and A2780cis cell line was examined. Cellular sensitivities for CP were determined by their IC_50_ values after 48 h of treatment. Fig 1A shows that CP significantly inhibited proliferation/viability of A2780 and A2780cis cells with IC_50_ values 12.3 μM and 28.8 μM respectively. The proliferation/viability of cells significantly decreased after 5 mM NaBu co-treatment with CP IC_50_ values 0.7 μM and 11.8 μM, respectively. To further determine dynamic of NaBu treatment we used sub-toxic doses of CP (5 μM) for 24, 48 and 72 h (Fig 1B). 5 mM NaBu alone significantly reduced proliferation/viability of A2780 and A2780cis cells after 48h (66.6 ± 12.8% and 62.0 ± 13.0%). CP (5 μM) alone decreased A2780 cell proliferation/viability to 65.6 ± 12.5% after 48 h, while being relatively ineffective due to the chemoresistance in A2780cis cells (84.3 ± 16.0%). The effect of CP treatment was pronounced when used in combination with NaBu. Proliferation/viability of A2780 and A2780cis cells was reduced to 32.0 ± 2.6% and 41 ± 12.1%, respectively. No effect of NaBu, CP or combination of both on proliferation/viability after 24 h of treatment was observed.

### Effect of sodium butyrate on cell cycle progression

**Fig 2:**
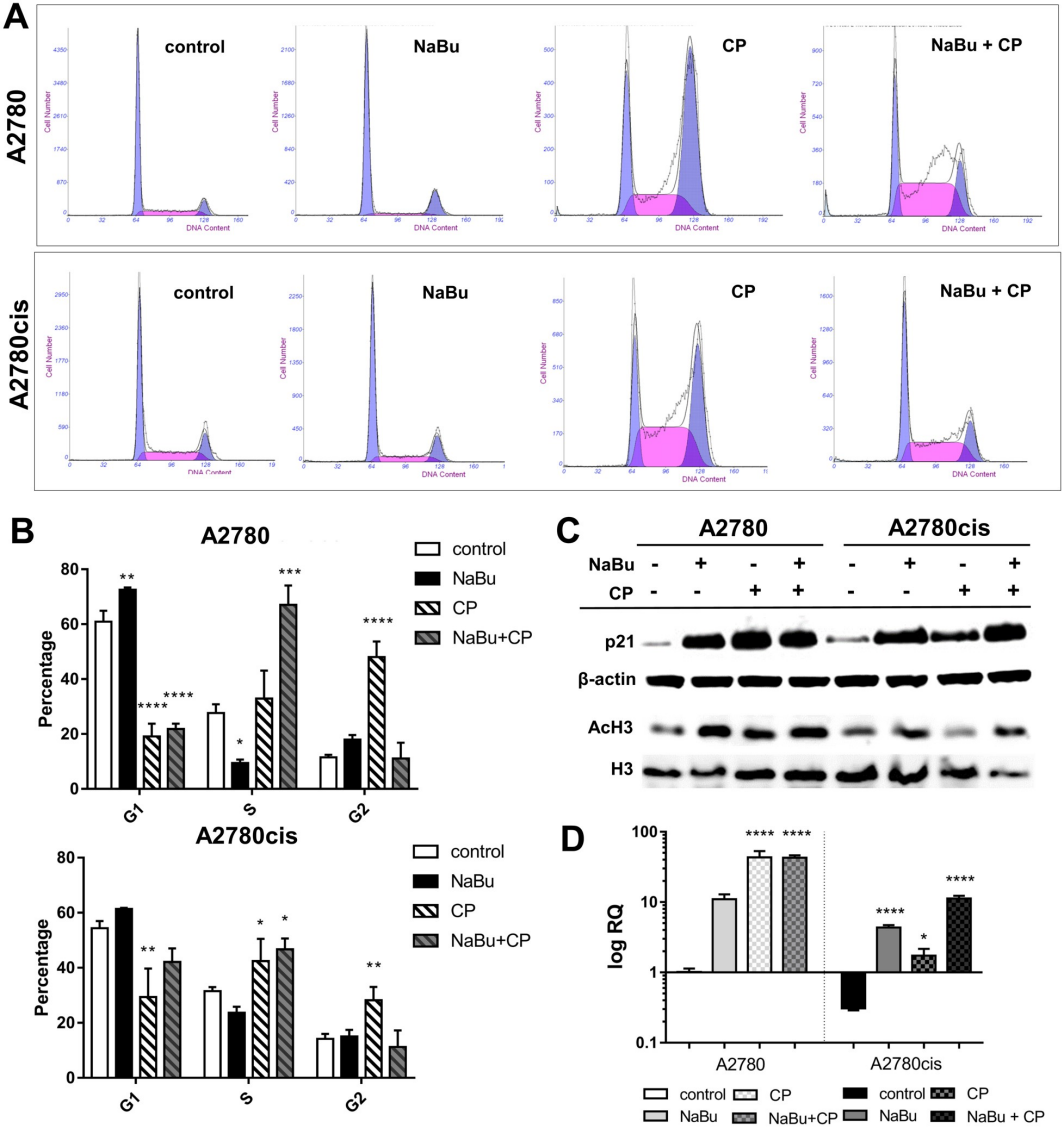
Effect of cisplatin and butyrate on the cell cycle and histone acetylation. Distribution of A2780 and A2780cis cells in G1, S, and G2/M phases of the cell cycle after 5 mM NaBu, 5 μM CP single-agent treatments and combination of both treatments was measured by flow cytometry (A, B). Panel A shows representative cell cycle analysis histograms. The bar graph (panel B) represents cumulative data on cell cycle distribution in the G1, S, and G2/M phase. The data points in the graph are the means ± SD of 3 independent experiments. Significance was tested using one-way ANOVA (*P ≤ 0.05, **P ≤ 0.01, *** P≤ 0.001, **** P≤ 0.0001 vs. control). p21, histone H3 and acetylated H3 protein amount was detected by immunoblot analysis (C). Blots shown are representative of at least three independent experiments. β-Actin was used as a loading control. Gene expression of *CDKN1* in A2780 and A2780cis cells was measured using qPCR (D). The results were normalized for the *HPRT* expression. Data are presented as means ± SD of 3 independent experiments. Significance was tested using one-way ANOVA. (*P ≤ 0.05, **P ≤ 0.01, *** P≤ 0.001, **** P≤ 0.0001 vs. control).

The effect of NaBu itself or in combination with CP on cell cycle progression was measured by flow cytometry (Fig 2A and 2B). NaBu exposure impeded the cell cycle transition from G1 to S phase in A2780 cells. The percentage of cells in G1 phase significantly increased from 60.9 ± 4.0% in control cells to 72.5 ± 0,8% in cells treated with NaBu for 24 h. These data correspond well with concomitant reduction in the S phase population, (27.6 ± 3,1% in control to 9.4 ± 1,3% in treated cells). NaBu treatment of A2780cis was accompanied by mild increase in the percentage of cells in G1 phase (54.3 ± 2.6% in control to 61.3 ± 0.4% in treated cells) and decrease in S phase (31.5 ± 1.5% to 23.6 ± 2.7% in treated cells). Exposure to CP (5 μM) arrested both A2780 and A2780cis cells in G2 phase with 48.1 ± 5.7% and 28.1 ± 4.9% respectively, compared to 11.5 ± 0.9 and 14.1 ± 1.8 in untreated controls. The cell cycle distribution profiles of cells treated with combination of CP and NaBu differed from that of cells treated with cisplatin or NaBu alone. NaBu was able to abrogate the G2 phase block induced by CP. When NaBu and CP were used in combination, there was massive accumulation of A2780 (67.1 ± 7.1%) and A2780cis (46.7 ± 3.9%) cells in S phase (control 27.7 ± 3.2, 31.5 ± 1.5 respectively).

The amount of cell cycle-regulating protein p21 was measured by Western blot (Fig 2C) and the expression of its gene *CDNK1* by qPCR (Fig 2D). Initial expression of *CDNK1* is lower in resistant A2780cis compared to non-resistant A2780 cells. However, we detected massive upregulation in p21 both on mRNA and protein level after NaBu, CP and NaBu+CP treatment in both A2780 and A2780cis cells. Resistant A2780cis cells showed milder increase in p21 expression after CP treatment in comparison to A2780 cells. Inhibition of HDACs activity was accompanied by histone H3 hyperacetylation as demonstrated by Western blot analysis. Significant increase in acetylation of histone H3 was observed when A2780 and A2780cis cells were incubated with 5 mM NaBu alone or in combination with CP.

### Profile of EMT-associated markers after sodium butyrate treatment

**Fig 3:**
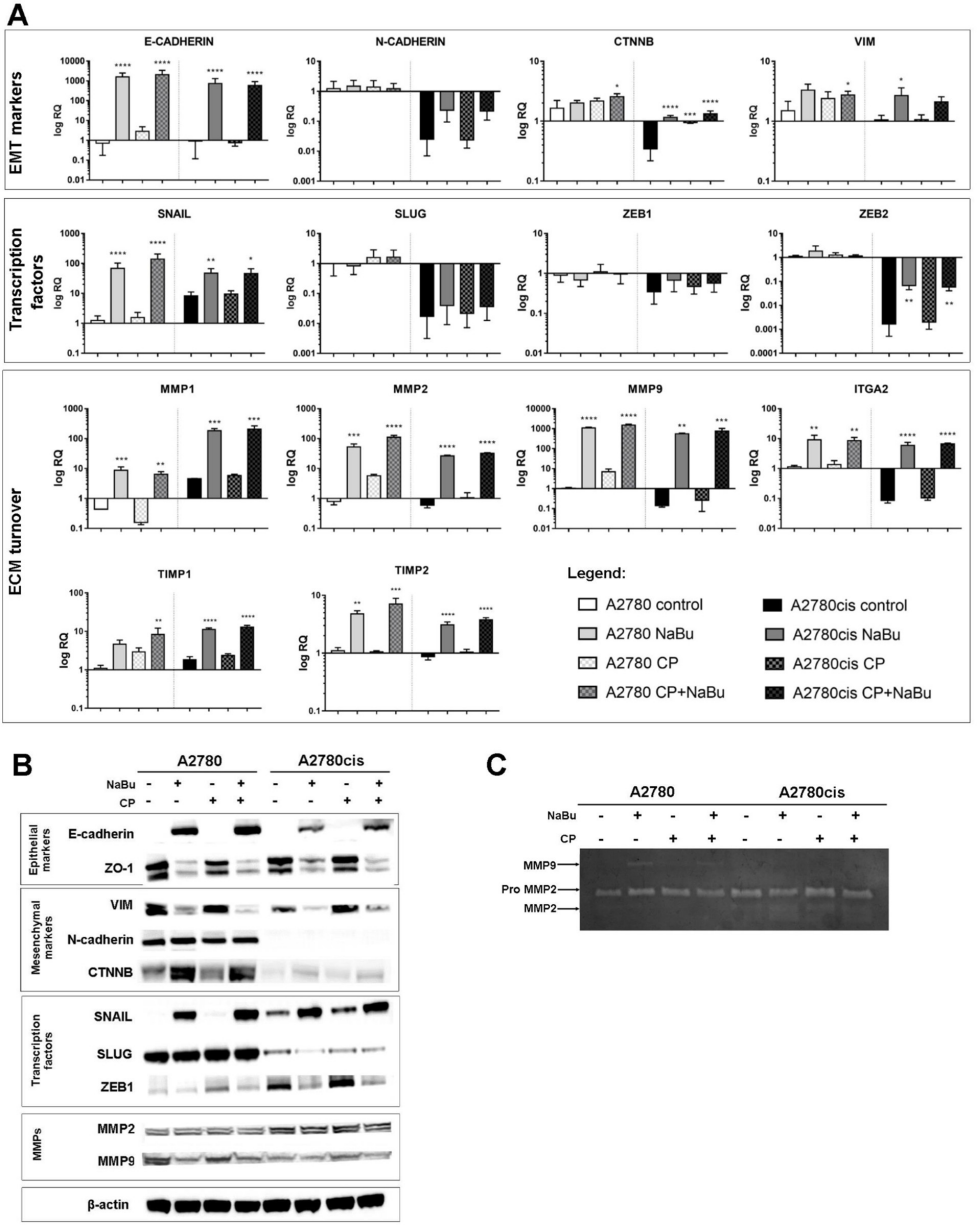
EMT related genes and proteins expression. Gene expression of EMT related genes in A2780 and A2780cis cells was measured using qPCR (A). Results were normalized for the *HPRT* expression. Data are presented as means ± SD of 3 independent experiments. Significance was tested using one-way ANOVA (*P ≤ 0.05, **P ≤ 0.01, *** P≤ 0.001, **** P≤ 0.0001 vs. control). Protein expression was detected by immunoblot analysis (B). A representative immunoblot of at least three independent experiments is presented. β-Actin was used as a loading control. Activities of MMP-2 and MMP-9 in the cell culture supernatants were measured by gelatin zymography. Gelatinolytic activity of pro and active MMP-2 and active MMP-9 are visible as a clear area on the gel (C).

To evaluate the effect of NaBu, CP or combination of both on the EMT-related genes expression we performed qPCR analysis. Fig 3A shows relative gene expression in A2780 and A2780cis cells after 24 h of NaBu (5 mM), CP (5 μM) alone or in combination. Chemoresistant A2780cis cells have lower basal expression levels of mesenchymal markers N-cadherin (*CDH2*), beta-catenin (*CTNNB1*), transcription factors SLUG (*SNAI2*), ZEB2 (*ZEB2*) gelatinase (*MMP9*) and integrin α2 subunit (*ITGA2*) than sensitive A2780 cells. The basal expression of collagenase (MMP1) and transcription factor SNAIL (*SNAI1*) is higher in A2780cis cells than in A2780.

Treatment of both cell lines with NaBu resulted in significant increase in epithelial marker E-cadherin (*CDH1* gene), as well as increase of mesenchymal marker vimentin (*VIM*) when compared to untreated control cells. Key EMT regulator transcription factor SNAIL (*SNAI1*), metalloproteinases MMP-1, -2, -9 (*MMP1*, *2*, *9*), tissue inhibitors of MMPs (*TIMP1*,*2*) and integrin α2 (*ITGA2*) were also increased. NaBu induced significant upregulation of beta-catenin (*CTNNB1*) and *ZEB2* transcription only in resistant A2780cis cells. With one exception, CP had no effect on gene expression of both cell lines. The combined treatment of NaBu and CP showed similar results to the NaBu alone treatment.

In order to further confirm the role of NaBu in regulation of EMT phenotype we performed western blot analysis (Fig 3B) to detect changes on protein level. Similar changes as seen in *SNAI1* (SNAIL) and *SNAI2* (SLUG) mRNA expression were found in immunoblot results: NaBu induced increase in SNAIL, but had no effect on SLUG. An increase in E-cadherin was demonstrated following NaBu treatment in both A2780 and A2780cis cells, which corresponds with changes in *CDH1* expression. Surprisingly the amount of another epithelial marker ZO-1 decreased. Mesenchymal marker vimentin was downregulated after NaBu treatment. Discrepancy with *VIM* mRNA expression suggests importance of post-transcriptional regulations. Both inactive and active MMP-2 forms were observed on Western blot with no differences in either groups in sensitive A2780 cells. Stronger expression could be observed in the A2780cis control and CP treated cell lysates. There was a decrease in the protein expression levels of MMP-9 in NaBu treated A2780 cells. The enzymatic activities of MMP-2 and MMP-9 in the serum-free cell culture supernatants were measured by gelatin zymography (Fig 3C). The gelatin zymogram showed a strong proteolytic band corresponding to latent pro MMP-2 in both cell lines. Active form of MMP-2 was detected in A2780cis. Mild increase after NaBu treatment was seen. Very weak gelatinolytic activity of 92-kDa MMP-9 was detectable in NaBu treated A2780 cells. Increased secreted MMP-9 activity correspond with lower intracellular protein levels in A2780 cells.

### CDH1 methylation

Our results showed no expression of E-cadherin on mRNA and protein level in both A2780 and A2780cis. NaBu caused drastic upregulation of this epithelial cell marker on both mRNA and protein level in both cell lines, therefore we investigated whether positive effect of NaBu treatment on E-cadherin (*CDH1*) expression was caused by reversal of its promoter methylation. Methylation level in 32 CpG sites in *CDH1* gene (promoter/exon1 regions, see S1 Fig) was compared in cisplatin-sensitive/resistant A2780 cell lines using bisulfite NGS. Cells were treated with NaBu, CP or combination of both for 24 h.

**Fig 4:**
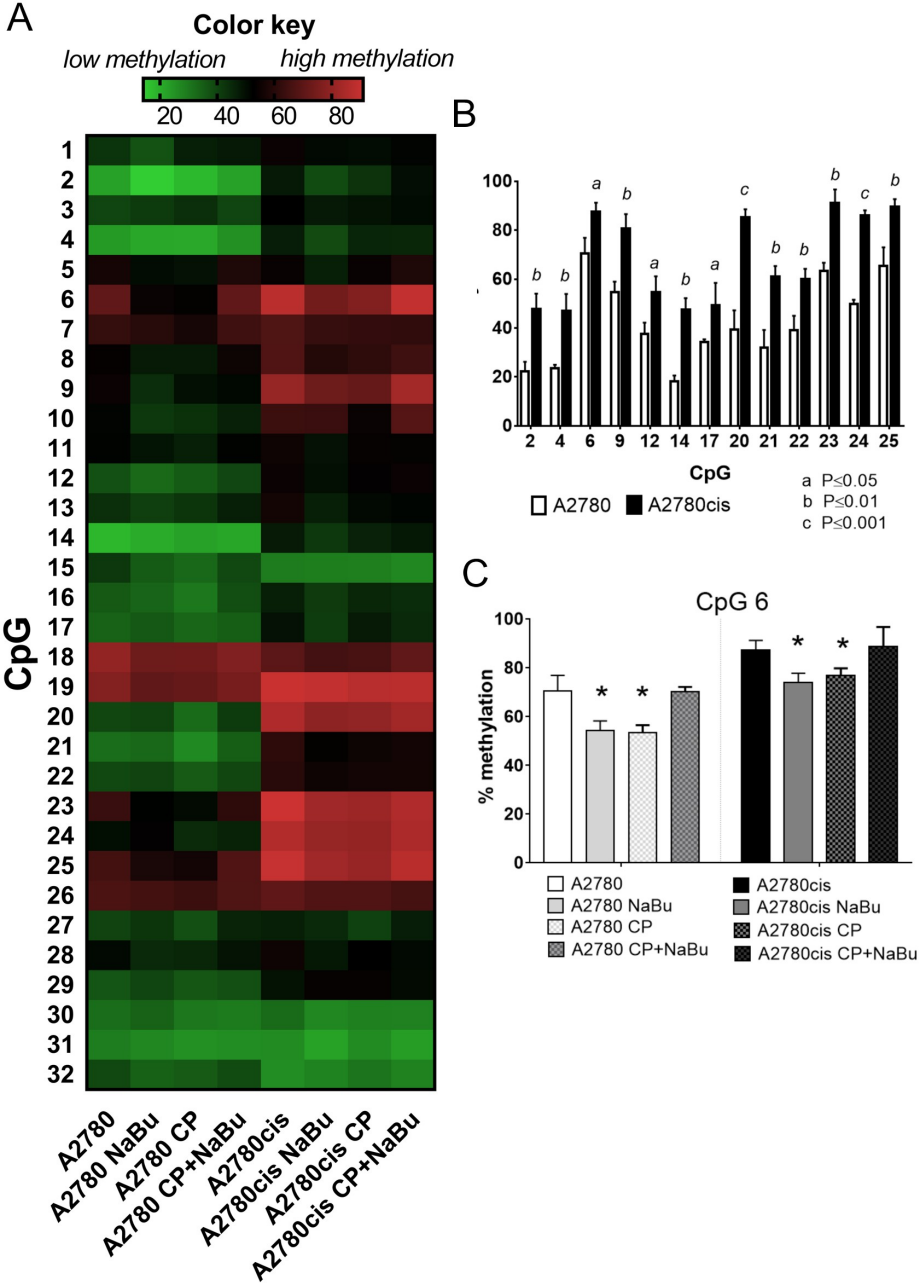
CDH1 gene methylation. Heat map displays changes in *CDH1* methylation in 32 CpG sites in A2780 and A2780cis cells treated with NaBu, CP or combination of both (A). Analysis was performed in three independent experiments, means are shown. Significantly hypermethylated individual CpG sites in A2780cis cells compared to A2780 cells (B). The effect of NaBu, CP or combined treatment on percentage of methylation at CpG site 6 (C). The plots represent means ± SD of 3 independent experiments. Significance was tested using Student t-test P ≤ 0.05.

As evident from the Fig 4A, overall *CDH1* methylation in cisplatin resistant cell line A2780cis is elevated compared to cisplatin sensitive cell line A2780. Fig 4B shows significantly hypermethylated CpG sites in A2780cis cells compared to A2780. NaBu did not significantly affect *CDH1* overall methylation either in A2780 cell line or in A2780cis cell line with exception of CpG 6, where significant demethylation was observed (Fig 4C).

## Discussion

Platinum-based drugs combined with taxanes are used as a gold standard of the ovarian cancer chemotherapy. Unfortunately, platinum-resistant recurrence frequently occurs; new therapeutic strategies are therefore intensively researched. It has been demonstrated that cancer and malignant transformation is not solely a result of genomic mutations, but that it is also driven by epigenetic alterations [14], such as DNA methylation or histone acetylation.

Recent evidence proves that expression of class I HDACs are increased in ovarian carcinomas and plays a significant role not only in carcinogenesis, but also in resistance to chemotherapeutic agents [15]. The correlation between HDACs overexpression and cisplatin resistance was also confirmed *in vitro* in SKOV3 and OVCAR3 epithelial ovarian cancer cell lines [16]. In our study we show that NaBu, a general HDAC inhibitor, decreases CP resistance of ovarian cancer cell line A2780cis. Moreover, our results show that NaBu induced changes in expression of genes critical for regulation of EMT.

In our study on A2780 and A2780cis ovarian cancer cell lines, combination treatments showed better therapeutic efficacy (based on IC_50_ calculations) than the individual drugs. This is in agreement with previously reported synergistic cytotoxic effect of NaBu and CP *in vitro* (HeLa cells) and *in vivo* tumor models [9]. The mechanism by which NaBu sensitized the cells towards CP seems to be associated with hyperacetylation of core histones, which induces relaxation of chromatin structure and makes DNA accessible to transcription factors. We observed that NaBu promoted histone H3 acetylation in A2780 as well as in A2780cis. We have shown that NaBu treatment inhibits proliferation of A2780 cells *in vitro* by arresting cells in G1 phase of the cell cycle and that this process correlates with increased expression of cyclin-dependent kinase inhibitor 1 –p21^WAF1^. HDACi-induced G1 arrest (including NaBu treatment) has been previously reported in ovarian carcinoma cell lines [17]. In our experiments CP blocked cell cycle progression at G2 phase with concomitant reduction in G1 phase in both cell lines and S-phase accumulation in A2780cis cell line. Although the same effect has been already described [18], exposure to CP in A2780 and A2780cis can result in a broad spectrum of effects on cell cycle distribution due to variations in exposition schedule [19, 20]. The results of our combination therapy are in contrast with those reported in the literature [9, 21]. When CP and NaBu were used in combination, S-phase arrested cells were prominent.

During oncogenesis, cell-cell adhesion is disturbed by genetic and epigenetic changes, resulting in changes in signalling, loss of contact inhibition, and altered cell migration and stromal interactions [22]. Epithelial—mesenchymal transition (EMT) is crucial in this process and thus implied in cancer progression and metastases. EMT has been also proposed to participate in chemoresistance development. Brozovic et al. [23] reported that ovarian cancer cell lines resistant to paclitaxel and cross-resistant to carboplatin (OVCAR-3/TP) are characterized with mesenchymal features compared to parental OVCAR-3 cell line. The process of EMT is dynamic and the cells that underwent EMT could re-acquire epithelial phenotype (mesenchymal—epithelial transition, MET). According to Tian et al. [24] different levels of miR-34, miR-200, SNAIL and ZEB enable to distinguish between epithelial, mesenchymal and hybrid cells. In our study cisplatin-resistant A2780cis exhibited mesenchymal phenotype due to moderate SNAIL and high ZEB1 protein expression, while sensitive A2780 cells were more epithelial (no SNAIL and very weak ZEB1 expression).

Recently it became apparent that epigenetic regulation of gene expression is crucial during EMT, although the current evidence on histone acetylation role in this process is rather contradictory. It was demonstrated that inhibition of HDAC alone can induce EMT in numerous epithelial cancer cell lines such as breast [25], prostate [26], colorectal [27] and head and neck [28, 29]. In these cells various HDACi induce hyperacetylation of histone H3 in promotor regions of pro-mesenchymal genes–*SNAI1*, *SNAI2*, *ZEB2*, *VIM*, which enhances their transcription and triggers EMT. On the other hand, others have demonstrated that during TGF-β induced EMT the activity of HDAC is required to trigger downregulation of epithelial marker E-cadherin and HDAC inhibitors can prevent TGF-β induced EMT. This was demonstrated mainly in nonmalignant cells of lens epithelium [30, 31], renal epithelial cells [32] or hepatocytes [33], but also in TGF-β challenged A549 cells derived from lung carcinoma [34]. However, the relationship between epigenetic histone modifications and EMT of ovarian cancer cells has not been studied so far. We have tested the effect of NaBu, CP or combination of both on chemosensitive A2780 and chemoresistant A2780cis ovarian cancer cells. We found that NaBu induced *CDH1* expression dramatically, whereas expression of VIM was decreased in both cell lines. NaBu-induced upregulation of *CDH1* could be caused by changes in methylation status of *CDH1* gene promotor. Gene regulation by CpG methylation is involved in a large spectrum of biological processes including ovarian cancer. Methylation of the *CDH1* was reported as a prognostic marker in ovarian clear cell and endometrioid adenocarcinoma [35]. Hypermethylation of *CDH1* promotor as well as transcriptional silencing was found in breast and ovarian cancer cell lines with inherent or acquired doxorubicin resistance [36]. Sarkat et al. [37] propose that HDACi can influence the initial methylation status of the DNA. Next generation sequencing approaches, that can provide robust DNA methylation analysis, are still relatively rare in the literature. Our study showed that the methylation degree of 10 out of 32 CpG sites in the *CDH1* gene promotor/exon1 regions is significantly increased in cisplatin-resistant ovarian cancer cells line A2780cis compared with sensitive A2780 cells. However, expression of *CDH1* did not reflect these changes, as mRNA and protein expression of E-cadherin was very low or non-detectable in both cell types. These findings support previous study [38], where hypermethylation of various genes in cisplatin-resistant A2780/cp70 was observed, but only small percentage of these hypermethylated genes were found to be functionally downregulated. NaBu-induced expression of *CDH1* was not connected with *CDH1* demethylation. It is therefore questionable whether promoter hypermethylation really plays a significant role in downregulation of E-cadherin gene expression in ovarian cancer cells.

In A2780cis one of *CDH1* repressors ZEB1 was downregulated after NaBu treatment. Surprisingly, we observed induction of another more potent repressor SNAIL. These contradictory results could be explained by the fact that *SNAI1* is not able to complete transcriptional repression of *CDH1* without the cooperation with *ZEB1*. Transcription factors SNAIL and ZEB1 act as a direct repressors of *CDH1* by binding its promotor [39, 40], but SNAIL initiates transcriptional repression of *CDH1* while ZEB is required for its completion [41]. Our results suggested that restoration of *CDH1* expression mediated by HDACi NaBu is SNAIL independent.

EMT is frequently associated with overexpression of matrix metalloproteinases collagenase (MMP1) and gelatinases (MMP2 and MMP9). Under physiological conditions low basal levels of MMP are found. MMP genes expression is primary regulated at the transcriptional level, proenzyme activation and endogenous inhibition by TIMPs. These regulatory mechanisms are lost in cancer cells. Moreover, recent evidence shows that chromatin remodeling plays a central role in controlling *MMP9* gene expression [42]. Correlation between MMP expression and invasiveness or metastatic tumors potential is very well documented. *MMP1* overexpression has been also reported in chemoresistant A2780 cell lines [43]. Diverse effects of various HDACi on MMP expression in cell lines have been reported [26, 44, 45]. Here we showed that treatment with NaBu had stimulatory effect on *MMP1*, *MMP2*, *MMP9* mRNA levels in both cell lines. But in the same time activates TIMP1, 2 expression to antagonize the proteolytic activity. Negligible changes in MMPs activities were observed after NaBu treatment.

Our findings support the hypothesis that HDACi treatment results in significant changes in gene transcription in ovarian cancer cells, which affects pathways critical for EMT. Significant differences were found in expression of EMT markers between CP sensitive A2780 and CP resistant A2780cis cells. NaBu sensitized both cell types to CP and upregulated epithelial marker E-cadherin. This effect was not associated with significant demethylation of promoter of E-cadherin gene *CDH1*. HDACi themselves or in combination with chemotherapeutics may have therapeutic applications, although the exact mechanisms of interplay between histone acetylations and altered expression of EMT proteins are far from understood. Heterogeneity and plasticity of EMT phenotype of cancer cells should be taken into account.

## Supporting information

S1 Table Primer sequences for the Bisulfite Next Generation Sequencing of CDH1. (TIF)

S1 Fig Mapping of the CDH1 gene position relative to the chromosome. Schematic representation of analyzed E-cadherin promoter/exon1 regions. Individual CpG sites are highlighted.
(TIF)
