# Supplementary figures and images for: The effect of sodium butyrate and cisplatin on expression of EMT markers
Source: PLoS One. 2019 Jan 17;14(1):e0210889. doi: 10.1371/journal.pone.0210889 (PMC6336326; doi:10.1371/journal.pone.0210889)

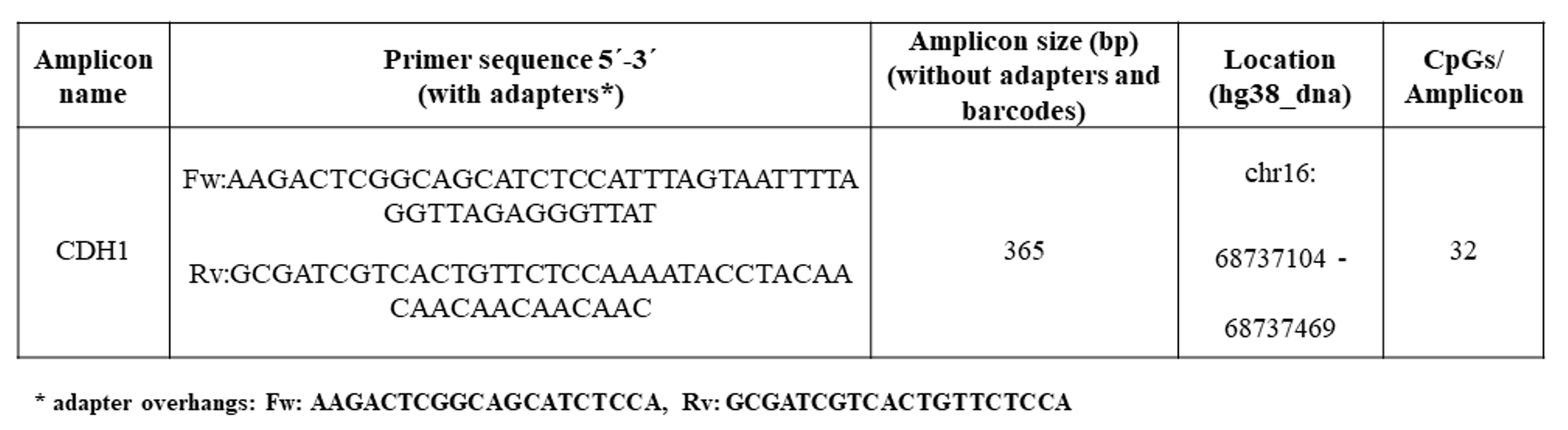

Supplement: S1 Table — (TIF) [file pone.0210889.s001.tif]
